# Drought stress in rice: morpho-physiological and molecular responses and marker-assisted breeding

**DOI:** 10.3389/fpls.2023.1215371

**Published:** 2023-07-18

**Authors:** Muhammad A. Hassan, Ni Dahu, Tong Hongning, Zhu Qian, Yi Yueming, Li Yiru, Wang Shimei

**Affiliations:** ^1^ Rice Research Institute, Anhui Academy of Agricultural Sciences, Hefei, China; ^2^ National Key Facility for Crop Gene Resources and Genetic Improvement, Institute of Crop Sciences, Chinese Academy of Agricultural Sciences, Beijing, China

**Keywords:** rice, drought, morpho-physiological responses, molecular responses, marker-assisted breeding, QTLs/genes

## Abstract

Rice (*Oryza Sativa* L.) is an essential constituent of the global food chain. Drought stress significantly diminished its productivity and threatened global food security. This review concisely discussed how drought stress negatively influenced the rice’s optimal growth cycle and altered its morpho-physiological, biochemical, and molecular responses. To withstand adverse drought conditions, plants activate their inherent drought resistance mechanism (escape, avoidance, tolerance, and recovery). Drought acclimation response is characterized by many notable responses, including redox homeostasis, osmotic modifications, balanced water relations, and restored metabolic activity. Drought tolerance is a complicated phenomenon, and conventional breeding strategies have only shown limited success. The application of molecular markers is a pragmatic technique to accelerate the ongoing breeding process, known as marker-assisted breeding. This review study compiled information about quantitative trait loci (QTLs) and genes associated with agronomic yield-related traits (grain size, grain yield, harvest index, etc.) under drought stress. It emphasized the significance of modern breeding techniques and marker-assisted selection (MAS) tools for introgressing the known QTLs/genes into elite rice lines to develop drought-tolerant rice varieties. Hence, this study will provide a solid foundation for understanding the complex phenomenon of drought stress and its utilization in future crop development programs. Though modern genetic markers are expensive, future crop development programs combined with conventional and MAS tools will help the breeders produce high-yielding and drought-tolerant rice varieties.

## 1 Introduction

Rice (*Oryza sativa* L.) is an important cereal crop consumed as a staple diet by half of the world’s population. Though it is grown globally, more than 50% of rice production was contributed by Asian countries (FAO, 2015; Donde et al., 2019a). It belongs to the Poaceae family, genus *Oryza*, containing 24 species, among which 22 are wild and 2 cultivated species (Gouda et al., 2020). The *O. glaberrima* and *O. sativa* were two well-known cultivated rice species, and their germplasm mainly originated from Asia, Europe, US, and African countries (Morishima, 1984). The *O. sativa* is the most widely grown rice species based on geographical adaptability. It is subdivided into *japonica*, *indica*, and *javanica* cultivars (Morishima, 1984). The *indica* and *japonica* varieties are primarily grown in tropical, sub-tropical, and temperate regions, while the *javanica* is a rare variety grown in hot and humid climates.

Rapidly changing climatic patterns have affected normal agricultural productivity and threatened global food security (Food and Agricultural Organization, 2020). The increasing human population has increased food consumption; increasing food demands required grain yield enhancements in cereal crops (rice, wheat, barley, maize) (Gouda et al., 2020). It is the need of the hour to develop rice cultivars compatible with climatic variations and sustain grain yield while curtailing the negative impacts of abiotic stress. Among all the abiotic factors, drought is the most detrimental, limiting almost 50% of rice productivity yearly(Bouman et al., 2005; Nelson et al., 2014; Pandey and Shukla, 2015). Drought is a meteorological phenomenon indicating insufficient rainfall or higher evaporation rates that cause water deficit conditions (Rollins et al., 2013; Upadhyaya and Panda, 2019). It is estimated that about one-third of the world’s total cropland suffers from drought stress; its intensity and severity are hard to predict as it depends on multiple factors such as rainfall frequency, evaporation rate, and soil moisture content (Rijsberman, 2006; Hao et al., 2018; Oladosu et al., 2019). Water availability below the optimum requirement compromised the actual yield potential of cultivars (Blum, 2011) by inhibiting active root growth and nutrient uptake (Todaka et al., 2017). Rice under drought stress exhibits poor growth and development due to inadequate morpho-physiological, biochemical, and molecular responses (Nahar et al., 2016; Kadam et al., 2017; Quinones et al., 2017). Breeding drought-tolerant varieties are a more sustainable and viable option for improving the rice’s capacity to withstand drought conditions and maintain its productivity potential (Pandey and Shukla, 2015).

Rice productivity is generally predicted by its agronomic attributes, such as the number of productive tillers, number of grains per panicle, 1000-grain weight, plant height, panicle length, grain size, and weight (Hua et al., 2002). These agronomic attributes are inherited and regulated through multiple genetic expressions. Most of the modern cultivar’s production potential remains stagnant because of the inability to cope with abiotic stress factors, i.e., drought, submergence, and salinity (Sabina et al., 2010). Plant breeders must develop genetically improved crop varieties that withstand biotic and abiotic stress conditions (Donde et al., 2019b; Gupta et al., 2019). Traditional breeding techniques are time-consuming and dependent on varying environmental conditions; breeding a new cultivar usually takes 8 to 12 years, and still, there are doubts about its authenticity (Sabina et al., 2010). Therefore, the breeder community is keenly interested in adopting modern breeding techniques and practices that simplify and speed up the breeding process. Pre-breeding techniques are employed for genetic mapping via the identification of genes/quantitative trait loci(s) (QTLs) that correspond to the phenotypic variation, after which these QTLs are introgressed into elite gene pools through marker-assisted selection (MAS). Modern technologies will speed up the varietal development procedure and generate diversified germplasm for future research. This article focused on the drought-induced implications and their mitigation by using MAS.

## 2 Responses to drought stress in rice

Rice productivity is extremely threatened under drought conditions. Drought-induced morpho-physiological damage and biochemical dysfunction are evident in rice plants, which curbs active plant growth and development. It is reported that drought stress affects rice yield by up to 90% depending on the intensity, duration, and crop growth stage (vegetative or reproductive) (Basnayake et al., 2006; Venuprasad et al., 2007). Crop plants tend to avoid, escape, tolerate, and recover from drought-induced implications; this phenomenon is collectively called drought resistance (Yue et al., 2006; Luo, 2010; Shanmugavadivel et al., 2019). We’ve provided a brief definition of these terms (drought avoidance, drought escape, drought tolerance, and drought recovery) here, even though they are interchangeably used in the context of drought resistance. Drought avoidance is characterized as the ability of plants to sustain high water potential and continue optimal plant growth under moisture-stress conditions (Kumar et al., 2017). Drought escape is the early completion of the plant growth cycle before the onset of local moisture deficit conditions (Manavalan et al., 2009). Drought tolerance refers to a plant’s innate capacity to survive in water-deficit conditions by sustaining physiological and biochemical activities with minimal plant damage (Luo, 2010). And the ability of a plant to restore its metabolic activity and regain its vigour after being exposed to extremely high levels of drought stress and dehydration is known as drought recovery (Luo, 2010). This section will briefly discuss how rice plants perceive drought stress and how their morpho-physiological and biochemical adjustments benefit survival mechanisms and maintain productivity (**Table 1**).

**Table 1:** Different drought resistance responses in rice under varying conditions of water-deficit stress.

Drought Responses	Escape	Avoidance	Tolerance	Recovery	References
**Morphological**	Quick growth Early flowering Early maturity Plasticity ↑	Leaf rolling & Glaucousness ↑ Deep rooting Limited vegetative growth Small sized leaves Waxy layer on leaf surfaces	Staying green for a longer duration Elasticity ↑ Primary & secondary root growth ↑	Temporary wilting of leaves Growth of new young leaves	(Srivalli et al., 2003; Basnayake et al., 2006; Yue et al., 2006; Luo, 2010; Du et al., 2011; Bennett et al., 2012; Rana et al., 2012; Upadhyaya et al., 2012; Devi and Kar, 2013; Das and Roychoudhury, 2014; Hu and Xiong, 2014; Kumar et al., 2017; Mangrauthia et al., 2017; Upadhyaya and Panda, 2019; Panda et al., 2021)
**Physiological**	Photosynthesis ↑ Respiration ↑	Stomatal closure Transpiration rate ↓ Water use efficiency ↑ Avoiding dehydration Water storage in plant organs ↑	Osmo-protectant accumulation ↑ Protoplasmic Resistance ↑ Stomatal conductance ↑ Sustaining photosynthetic activity Dehydrant enzymatic activity ↑ Hydraulic conductivity ↑ ROS scavenging	Temporary loss of turgidity ROS detoxification ↑ Antioxidant activity ↑ Water conductance resumed	
**Biochemical**	Leaf N level ↑		Osmotic adjustments Accumulation of soluble sugars ↑ Proline content ↑	Utilization of stored carbohydrates ↑	

Here “↑” indicates increasing rate, while “↓” shows decreasing rate.

### 2.1 Morphological and yield-associated responses

Unlike other cereals, rice is a water-loving plant susceptible to drought stress (Panda et al., 2021). According to Fukai and Cooper (1995), roots, shoots, and leaves’ responses to drought vary depending on the plant growth stage (early seedling, vegetative, or reproductive), the intensity of drought stress (mild to severe), and other environmental conditions. Various morphological parameters have been used to monitor plant responses to drought stress (Zaher-Ara et al., 2016; Upadhyaya and Panda, 2019). Drought stress alters the anatomy and ultrastructure of the leaf (Upadhyaya and Panda, 2019). Drought-induced low-water potential limits leaf growth (Zhu et al., 2020); additionally, reduced leaf area, leaf rolling, wilting, thickened leaf size, early senescence, stomatal closure, and cutinized layer on the leaf surface are some of the morphological traits associated with drought stress (Mishra and Panda, 2017; Hussain et al., 2018; Panda et al., 2021).

Root system efficiency is vital in combating drought stress conditions (Panda et al., 2021). Comas et al. (2013) stated that root mass (dry) and length are used to forecast rice production under water stress. Rice cultivars with a deep and prolific root system perform better in drought conditions (Mishra et al., 2019; Kim et al., 2020). Extreme drought conditions limit secondary root growth, thicken primary root structures, disrupt water relations, and result in poor nutrient uptake, leading to poor or stunted plant growth (Hussain et al., 2018; Panda et al., 2021).

**Figure 1:**
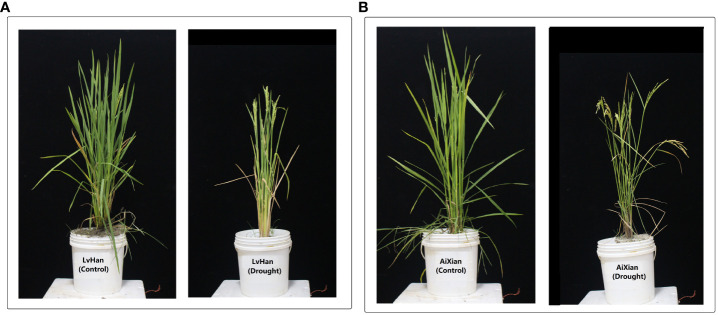
The field experiment exhibited the effects of drought stress on two rice cultivars, LvHan and AiXian (LvHan: drought tolerant and AiXian: drought sensitive), which are evident when compared to the control treatments. **(A)** LvHan (Drought) is subjected to drought stress at the early heading stage, and LvHan (Control) undergoes well-watered conditions throughout the growth cycle. **(B)** AiXian (Drought) is subjected to drought stress at the early heading stage, and AiXian (Control) undergoes well-watered conditions throughout the growth cycle. The experiment was grown in field conditions; drought stress was imposed at the heading stage. For drought treatments, irrigation stopped till leaves started wilting, and sprinkler irrigation resumed till harvesting maturity. Photos were taken after the flowering stage. (Unpublished: Own Experiment).

**Figure 2:**
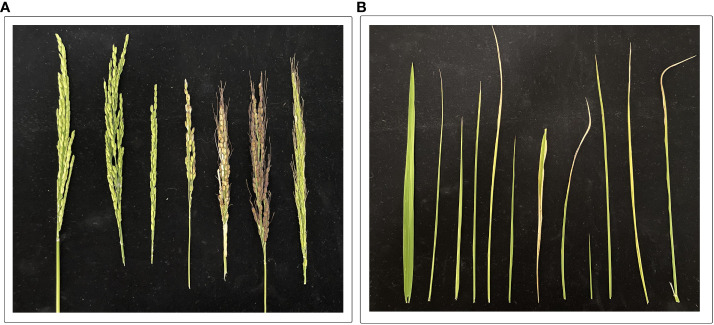
It is evident that the effects of drought stress on **(A)** panicles (starting from left side: two panicles are control treatments) and **(B)** flag leaves (starting from left: first leaf is control treatments) in various rice lines. The experiment was grown under field conditions; drought stress was imposed at the heading stage. For drought treatments, irrigation stopped till leaves started wilting, and sprinkler irrigation resumed till harvesting maturity. Photos were taken after the flowering stage. (Unpublished: Own Experiment).

**Figure 3:**
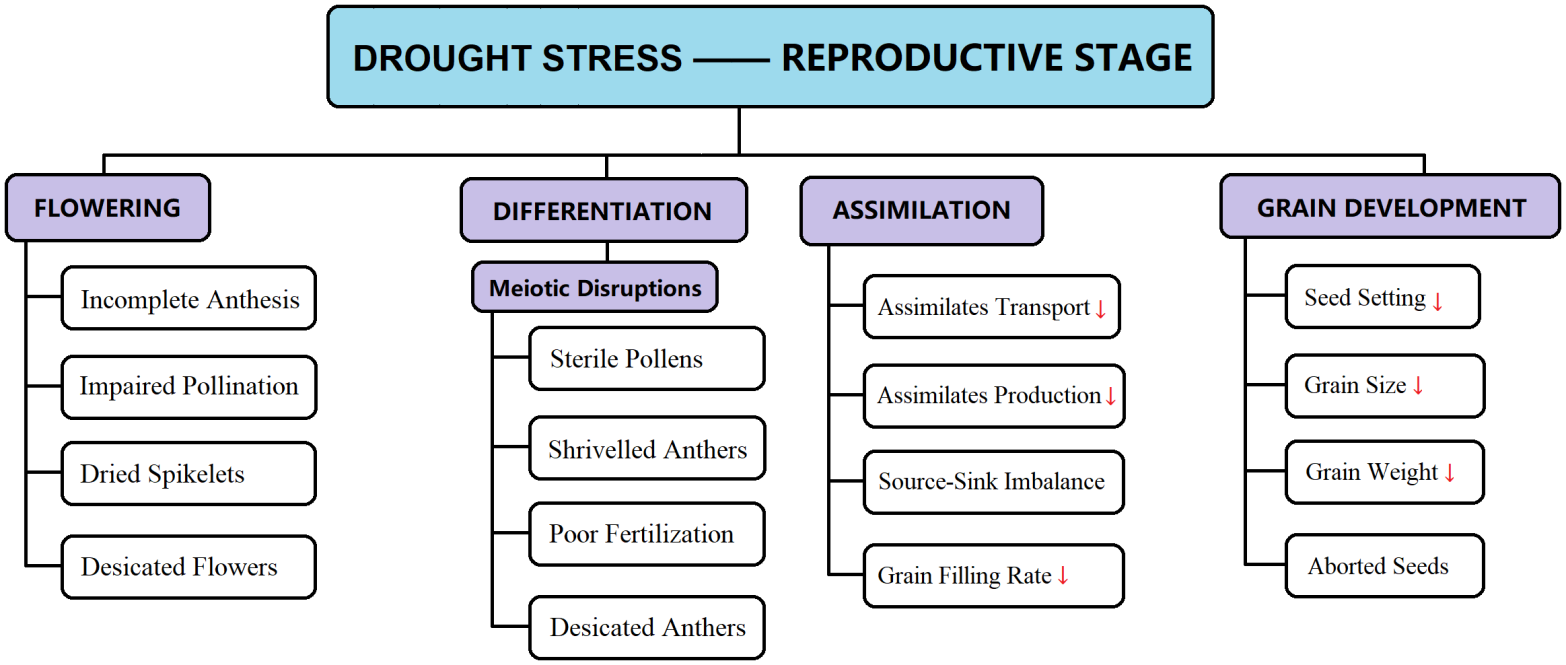
Drought-induced reproductive abnormalities are briefly illustrated for specific growth stages (e.g., flowering, differentiation, assimilate transport, grain filling, and so on). These disruptions in normal growth substantially decline the final paddy yield. [Here “↓” indicates decreasing rate].

Rice has three sensitive growth stages concerning drought stress: early seedling, vegetative, and anthesis (reproductive) (Singh et al., 2012). Water scarcity in the early seedling stage reduces drought stress, leading to unbalanced and poor stand establishment (Vibhuti et al., 2015). Drought stress interrupted active seed germination, causing osmotic imbalance, membrane impairment, decreased respiration, and ATP production (Kadam et al., 2017). Water constraint during the vegetative period causes delayed panicle initiation, followed by late maturity (Lilley and Fukai, 1994; Singh et al., 2012), directly correlated with yield decline. The most damaging impact of drought stress on grain yield appears to be during the reproductive growth stage. However, plants tend to recover during the vegetative growth phase, but recovery from the drought stress during the flowering phase is more complicated (Pantuwan et al., 2002; Alam Khan, 2012; Xangsayasane et al., 2014). A short span of drought stress during the reproductive growth phase severely curbs the rice grain yield by diminishing panicle length, poor seed setting, reduced number of kernels per panicle, and poor spikelet development (**Figures 1**–**3**) (Sikuku, 2012; Wei et al., 2017). It has been reported that drought stress during flowering has a detrimental impact on pollination, resulting in poor seed setting and reduced grain size and grain number; in severe drought cases, flowers abortion takes place, leading to a 100% yield decline (Hsiao et al., 1976; International Rice Research Institute, 1995; Kumar et al., 2006; Davatgar et al., 2009). It is therefore established that any intensity of drought stress (mild or severe) during the reproductive growth phase lowers the final grain production; this is because the translocation of assimilates from leaves to reproductive organs (panicle, kernel) is interrupted (Rahman et al., 2002). Additionally, rice cultivars that recovered from temporary drought patches exhibited better yield responses than drought-sensitive cultivars (Singh et al., 2012).

### 2.2 Physiological and biochemical responses

**Figure 4:**
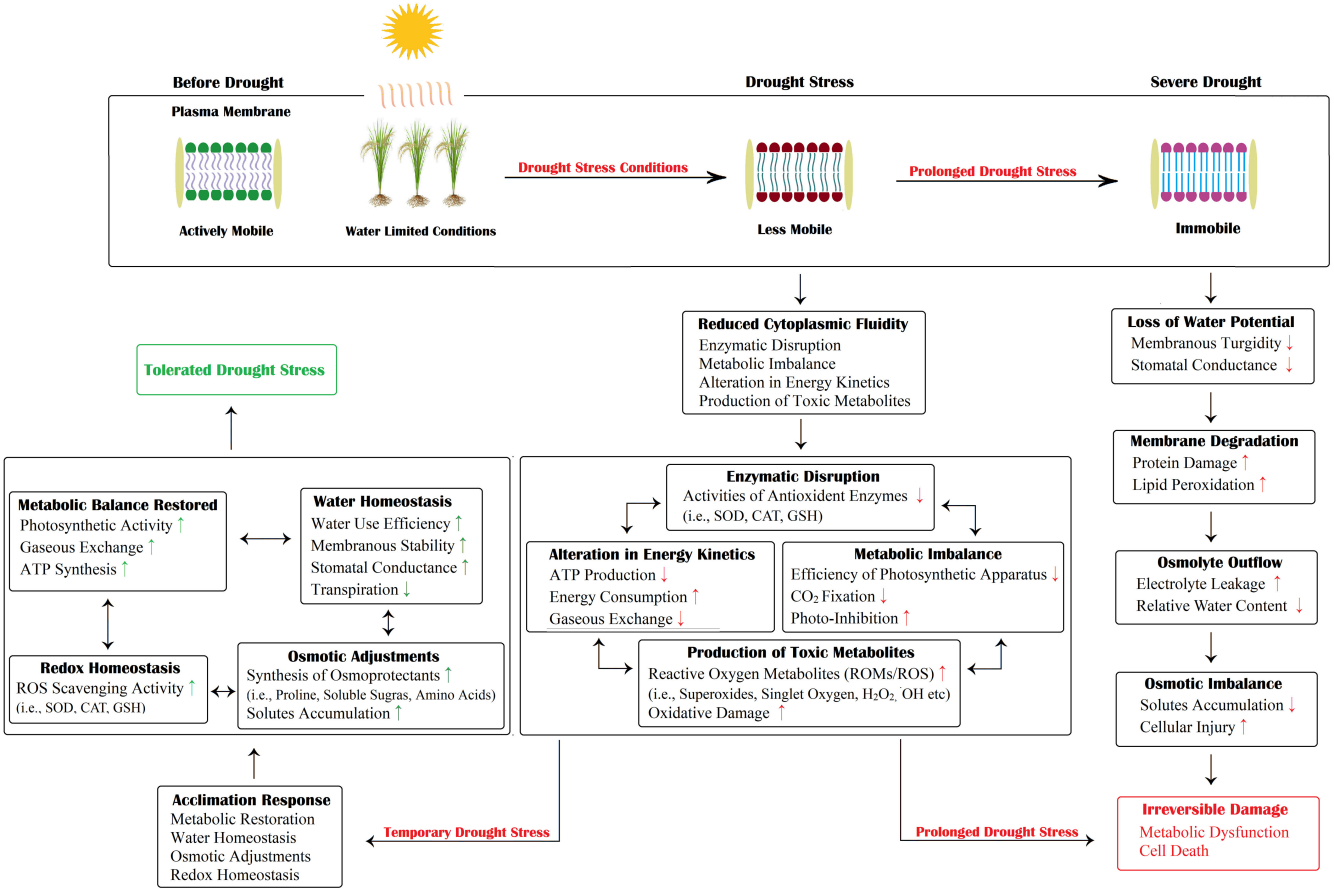
Schematic illustration of drought-induced damage and acclimation responses. The plant cell undergoes a number of osmotic, enzymatic, and metabolic changes. However, a severe and prolonged drought may cause irreparable damage to plant cells, eventually resulting in cell death. In contrast, short-duration drought stress can be tolerated. The plant resumes its normal growth and functioning. Here, “↑” and “↓” indicates a positive increase/decrease in a certain activity while “↑” and “↓” indicates a negative increase/decrease in a certain activity.

Drought stress disrupts the normal physiological functioning of rice plants, followed by restricted growth and reduced productivity (Upadhyaya and Panda, 2019). Drought-induced malfunctioning of vital physiological processes includes diminished photosynthetic activity, decreased water use efficiency (WUE), low transpiration rate, poor stomatal conductance, reduced CO_2_ concentration, imbalanced water relations and membrane impairment (**Figure 4**) (Dash et al., 2018; Zhu et al., 2020; Panda et al., 2021).

#### 2.2.1 Water-relations

Plant water relations are attributed through various terminologies such as leaf water potential, relative water content (RWC), turgor pressure, WUE, and membrane stability index. The RWC and WUE are crucial metrics for determining rice’s yield potential and performance in drought conditions (Farooq et al., 2009; Panda et al., 2021). Plant RWC is negatively impacted by moisture stress conditions, followed by osmotic imbalance, water exclusion, lipid peroxidation, membrane damage, and necrosis (Rao and Chaitanya, 2016). Drought-tolerant rice varieties can maintain adequate RWC and prevent membrane impairment. In a research investigation, Choudhary et al. (2009) exposed 28 days rice seedlings to water-scarce conditions for 72 hours. The results exhibited a uniform increase in RWC through osmotic adjustments (increased proline synthesis) and prevented membrane damage. The ability of crop plants to maintain membrane stability under drought-stress conditions is a vital feature of their tolerance mechanism (Pandey and Shukla, 2015). The Membrane stability index has been studied for its correlation with rice yield under drought conditions (Upadhyaya and Panda, 2019).

#### 2.2.2 Photosynthesis

Photosynthesis (PS) is a vital physiological process that largely accounts for dry matter production in crop plants. Water-scarce conditions negatively influenced the status of RWC in plants; as water-saving tactics, stomatal closure occurs, reducing CO_2_ influx, decreased transpiration rate, poor gaseous exchange, and electron transport. Drought stress lowers the efficiency of photosystems I & II (PSI and PSII), impairs rubisco activity and inhibits electron transport chain and ATP production (Farooq et al., 2009; Mishra and Panda, 2017; Upadhyaya and Panda, 2019). Photosynthetic pigments (i.e., chlorophyll, phycobilin, and carotenoids) showed lower efficiency under water-deficient conditions, resulting in inadequate light absorption, reduced light harvesting, and poor quality photoprotection (Jahan et al., 2013). Eventually causes limited photosynthesis and restricts photosynthates’ production (Fahad et al., 2017; Gupta et al., 2020). Carotenoids also serve as a precursor for plant signaling under stress conditions, so a decrease in carotenoid contents has a negative impact on signal perception during drought stress (Ashraf and Harris, 2013; Panda et al., 2021).

#### 2.2.3 Nitrogen metabolism

Nitrogen (N) metabolism is handy in combating water-deficient stress conditions, particularly in plants’ tolerance response (Suresh et al., 2015). Plants tend to regulate nitrogen metabolism under drought stress conditions by decreasing nitrate reductase activity (Xu et al., 2015). Glutamate dehydrogenase (GD), a stress-responsive enzyme, is vital in N metabolism and highly effective in detoxifying intracellular ammonia, synthesizing proline, and producing glutamate and soluble sugars (Zhou et al., 2015). Zhong et al. (2017) conducted an experiment in which rice plants were exposed to water deficit conditions with treatments of varying N levels. According to this study, high N levels increase rice’s ability to adapt to water stress by reducing stomatal restrictions on photosynthesis, maintaining higher Rubisco activity, and enhancing the assimilation of nitrate and ammonia. Another experimental study conducted by Cao et al. (2018) confirmed that nitrogen fertilization in rice improved the rice’s ability to withstand drought conditions by influencing photosynthetic activity, hormonal balance, carbohydrate assimilation, and distribution to other plant parts.

#### 2.2.4 Mineral homeostasis

Plant drought tolerance responses are mediated by balanced mineral nutrition. These vital minerals, such as nitrogen, silicon, magnesium, calcium etc. are taken up by plants through their roots via water absorption. The uptake of these essential elements is pivotal in balancing a plant’s mineral homeostasis and acclimation responses to abiotic stresses (Waraich et al., 2011; Zhong et al., 2017). Drought limits the active uptake of these essential minerals, resulting in stunted plant growth (Upadhyaya et al., 2013; Upadhyaya and Panda, 2019). It is reported that silicon fertilization increases the rate of photosynthesis, mineral absorption, and water use efficiency in rice, which helps counter the implications of drought stress more effectively (Chen et al., 2011; Cooke and Leishman, 2016). It has been reported that silicon and selenium effectively combat drought adversities by increasing the contents of amylase, phenolics, carbohydrates, and proteins, thereby increasing final grain yield (Emam et al., 2014; Suh et al., 2015). Li et al. (2017) reported the role of aquaporins in regulating hydraulic conductivity and its assisted by nitrate nutrition. Hydraulic conductivity is critical because of its role in facilitating plant nutrient transportation. Potassium (K) is also considered an essential nutrient having indispensable roles in plant physiology. It regulates the plant water potential and facilitates alleviating drought stress in tobacco and rice (Ahmad et al., 2016; Chen et al., 2017). Calcium (Ca) also effectively mitigates drought repercussions, particularly post-drought recovery responses (Devi and Kar, 2013). Zinc (Zn) nutrition is handy in ameliorating drought stress responses and post-drought recovery (Upadhyaya et al., 2017; Upadhyaya et al., 2020). It is directly and indirectly involved in various plant physiological activities, but any deviation from the optimum level results in toxicity and alterations in plant cell physiology, biochemistry, and anatomy (Alloway, 2013; Billard et al., 2015; Mattiello et al., 2015). The severe deficiency of Zn causes the disintegration of the cell membrane that hinders active plant growth and significantly dents the final grain yield; therefore, Zn fertilization is an effective way to overcome drought-induced complexities (Upadhyaya et al., 2013; Upadhyaya et al., 2017). Increased reactive oxygen species (ROS) accumulation under drought damages cellular structures because it interacts with lipids, proteins, nucleic acids, and pigments, impairing membrane function and causing lipid peroxidation that compromises cell viability. It can be avoided by increasing the scavenging response by antioxidant enzymes (Bartels and Sunkar, 2005; Pandey and Shukla, 2015). Under water-scarce conditions, the recommended fertilization of macronutrients (N, P, K, and Ca) and micronutrients (Si, Zn, and Mg) requires for activation of antioxidant defense mechanism and protection of plant cells from the harmful consequences of ROS accumulation (Dimkpa et al., 2017; Khan et al., 2017). Boron (B) is also thought to play a role in drought tolerance responses by promoting seed germination, mediating sugar transport, maintaining flower architecture, and developing pollen (Waraich et al., 2011). Recent research studies revealed that employing nano-fertilizers (particularly for micro-nutrients) in paddy fields will effectively tolerate the detrimental impacts of drought stress (Adhikari et al., 2016; Liu et al., 2016).

#### 2.2.5 Osmotic adjustments

Low precipitation and dry conditions undermine plant turgidity. In water-deficit conditions, plants maintain their turgor by accumulating osmolytes, i.e., proline, soluble sugars (SS), amino-acids, and phenolics (Anjum et al., 2011); this phenomenon is called osmoregulation. Proline is a type of amino acid used as a protein building block in plants, considered a vital osmoprotectant (Hayat et al., 2012). Increased proline content was first observed in ryegrass under water-scarce conditions (Kemble and Macpherson, 1954). Mishra et al. (2018) found increased proline accumulation in rice under water deficit conditions compared to normal irrigated conditions. Increasing proline content is directly related to drought tolerance as it helps the plant continue stomatal conductance and maintain leaf turgidity (Kumar et al., 2017). Soluble sugars are critical for optimizing various physiological functions, notably photosynthesis and mitochondrial respiration (Gill and Tuteja, 2010a). Soluble sugar accumulation under drought protects cell membrane integrity and acts as an osmoprotectant (Upadhyaya and Panda, 2019; Hassan et al., 2021). There were rare studies conducted to investigate how soluble sugars relieve drought stress.

#### 2.2.6 ROS accumulation and scavenging

Initially, ROS was found as a natural byproduct of aerobic metabolism; later also identified their role as a secondary signaling messenger under various environmental stresses (Panda et al., 2021). The excessive production of ROS and electrolytic leakage cause an imbalance in cellular homeostasis leading to oxidative damage to plant cells. Its severity may lead to cell death (Gill and Tuteja, 2010b). Thus, ROS is a double-edged sword since it causes oxidative damage when undergoing different abiotic stresses (i.e., drought, salinity, heavy metal stress, etc.). On the other hand, it acts as a signaling molecule in various physiological activities such as stomatal conductance, leaf senescence, and root hair growth (Das and Roychoudhury, 2014). ROS’s dynamic equilibrium is needed to regulate active physiological functioning and optimal plant growth. Moisture stress in paddy fields causes ROS production and scavenging imbalance, leading to membrane impairments, degradation of biomolecules (such as proteins, lipids, and DNA), and disruption in physiological processes (Bartels and Sunkar, 2005; Gill and Tuteja, 2010b).

ROS scavenging has been accomplished by activating the antioxidant defence mechanism through the antioxidant enzymes and non-enzymatic antioxidant components (Das and Roychoudhury, 2014). The antioxidant enzymes are superoxide dismutase (SOD), catalase (CAT), guaiacol peroxidase (GPX), ascorbate peroxidase (APX), glutathione reductase (GR), dehydro ascorbate reductase (DHAR) and monodehydroascorbate reductase (MDHAR). Non-enzymatic antioxidant components include ascorbic acid (AA), glutathione (GSH), α-tocopherol, carotenoids, flavonoids, and the osmolyte proline. The efficient antioxidant defence mechanism will be vital in countering the drought-induced repercussions of oxidative damage in rice (Mishra and Panda, 2017). SOD is found in almost all cellular organelles, and under drought-induced oxidative stress, increased SOD activity has been observed in rice plants (Melandri et al., 2020). CAT is found in mitochondria and peroxisomes, and its enhanced or diminished activity depends on stress intensity, it directly dismutases the H_2_O_2_ into H_2_O and O_2_ (Gill and Tuteja, 2010b). GPX is a well-known ROS scavenger and produces various related compounds, such as lignin, pyrogallol, and guaiacol, which act as scavenging donors for H_2_O_2_. Mishra and Panda, 2017 stated that rice’s GPX level increased under drought conditions. There are also many non-enzymatic biochemicals (such as proline, glycine betaine, A-tocopherol, ascorbic acid, carotenoids, flavonoids, glutathione etc.) which protects plant cell from drought induced adverse impacts of oxidative stress via minimizing the harms of ROS (Hussain et al., 2019). Proline is known as prominent inhibitor against the programmed cell death due to oxidative stress (Aslam et al., 2022). Glycine betaine is considered as major osmolyte which maintains the membranous integrity under unfavorable environmental conditions. Similarly, tocopherols, ascorbic acid are present in the thylakoids of chloroplasts and meristematic cells, respectively, protects the lipid peroxidation and responsible for membranous stability through quenching ROS damaging effect (Hussain et al., 2019); an important metabolite, glutathione, is essential for scavenging ROS to prevent oxidative damage in all physiological compartments of plant cell (Yaqoob et al., 2022). Ascorbate-Glutathione (AsA-GSH) mechanism is vital in eliminating excessive H_2_O_2_ from rice plants (Wang et al., 2012; Bhattacharjee and Dey, 2018). Various studies elucidated the significance of the AsA-GSH cycle in countering the water-scarce-induced drought responses in rice (Qureshi et al., 2018; Melandri et al., 2020). According to Nahar et al. (2016), rice cultivars that are tolerant of drought stress produced more antioxidants, followed by activation of the antioxidant defence system, than those that were drought sensitive, leading to oxidative stress and plant death in severe cases.

### 2.3 Molecular responses

**Figure 5:**
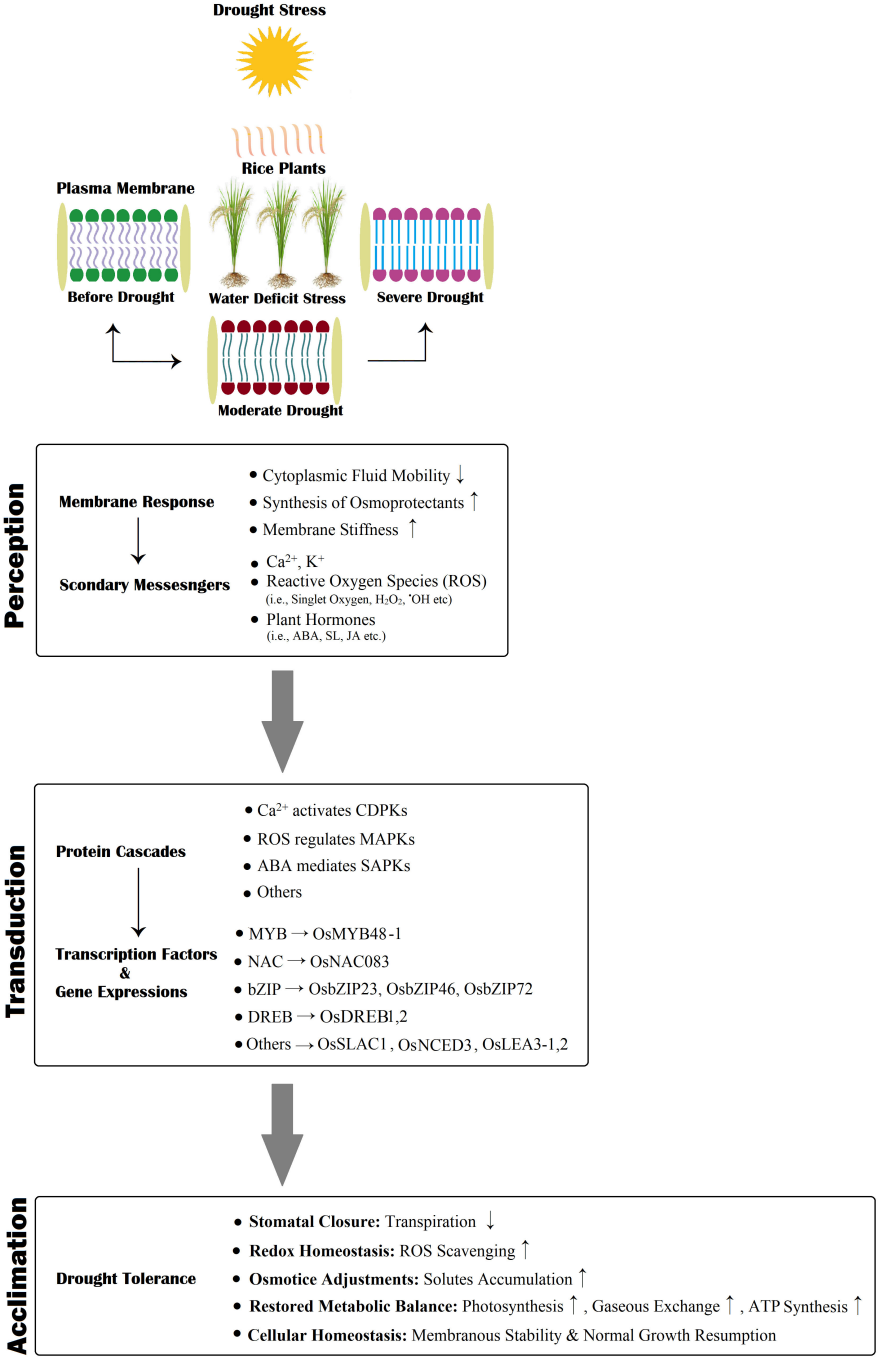
A schematic representation of rice drought perception, transduction, and final counter-response (acclimation). Drought stress is initially detected at the plasma membrane; subsequently followed by the influx of receptors, i.e., Ca 2+, ROS, Phytohormones, the initiation of protein kinase cascades, and protein cascade-driven up/downstream regulation generate gene expressions that assist drought tolerance. Here, “↑” indicates enhanced activity, and “↓” shows diminished activity.

Rice exhibits a diversified molecular response to drought stress (**Figure 5**). The drought tolerance mechanism is initiated with signal sensing, followed by signal perception, transduction, genetic expressions, cellular regulation, and survival metabolic responses (Du et al., 2011; Oladosu et al., 2019). Drought is a multifaceted abiotic condition acclimated through regulating numerous genetic expressions (Kumar et al., 2017). Rice exposure to water-deficit stress exhibited multiple differential gene expressions, with about 5000 up-regulated and 6000 down-regulated gene expressions (Bin Rahman and Zhang, 2016; Joshi et al., 2016). These genes are categorized based on their localized functioning: (1) genes associated with membrane transport, (2) genes involved in signaling, and (3) transcriptional regulation (Kim et al., 2020). These genetic expressions are responsible for most rice plants’ drought-induced physiological, biochemical, and molecular acclimation responses(Dash et al., 2018; Gupta et al., 2020). Transcriptomic and proteomic studies on rice have identified the transcriptomic factors (i.e., *MYB*, *DREB/CBF*, *AREB/ABF*, *NAC*, etc.) and their role in regulating the transcription of drought inducive gene expressions(Nahar et al., 2016; Zhang et al., 2016). Kumar et al. (2012) also reported the number of gene expressions and transcription factors (TFs) responsible for the drought tolerance response in rice. Previous research studies advocated that there are two main regulatory pathways for the induction of gene expression patterns for drought resistance mechanisms, known as (1) ABA-dependent and (2) ABA-independent regulation pathways (Du et al., 2011; Fu et al., 2017). The *MYB*, *NAC*, and *AREB/ABF* TFs drive the ABA-dependent pathway, while ABA-independent pathways are regulated via *DREB* TFs. Rabbani et al. (2003) stated that the exogenous application of ABA in rice effectively induces genetic expressions for combating the negative impacts of drought stress. A study on upland rice reported the role of drought-responsive genes in various signaling pathways (i.e., Ca^2+^, ABA, and ethylene-accompanied proteins kinases and inducive factors), reducing oxidative damage, maintaining cellular homeostasis and osmoregulation (Rabello et al., 2008). The ABA-receptor complex regulates ABA-responsive transcription through *AREB/ABF*, and it involves *SnRK2*, which is integral for activating *ARB/ABF* by phosphorylation (Umezawa et al., 2010). The function of *SnRK2* indicates the significance of the plant’s drought-responsive mechanism via swift adaptive action by plants under stress (Upadhyaya and Panda, 2019). As we mentioned earlier, ABA-independent pathways are governed by *DREB* TFs. Transcription of various genetic expressions in plant tissues is activated by the *DREB* TFs (Upadhyaya and Panda, 2019). The TF, *C2H2-type*, regulates stomatal closure upon exposure to water-deficit stress; this TF is also responsible for the induction of gene expression for quenching ROS and H_2_O_2_ and maintaining their dynamic balance under drought stress (Huang et al., 2009). Cominelli et al. (2005) stated that the expression of TFs, *AtMYB60* and *AtMYB60* primarily found in guard cells and controls the opening and closing of the stomatal aperture under drought tolerance responses.

## 3 Modern breeding with marker-assisted selection for drought management

Currently, many significant efforts are in progress to develop drought-resilient rice cultivars. Modern breeding techniques identify genes/QTLs regarding their phenotypic traits. After mapping, they are introduced into an elite gene pool, followed by MAS and the development of drought-tolerant varieties. The MAS is an indirect selection method in which the desired attributes are chosen based on markers (morphological, biochemical, or DNA/RNA variation) associated with a particular desired characteristic, i.e., high yield, drought resistance, tolerance (Lamont et al., 2014). Employing biomarkers and modern breeding approaches (i.e., molecular breeding and gene editing tools) is highly recommended to speed up the varietal development process (Donde et al., 2019b; Gouda et al., 2020). These modern approaches are very effective in discovering and better understanding the complex biological mechanisms in rice plants, i.e., drought tolerance, salt tolerance, cold tolerance etc. (Shakiba et al., 2017; Oladosu et al., 2019). Drought tolerance is a multifaceted mechanism operated through various genes and QTLs (Suh et al., 2015). These genetic variations can be utilized to develop drought-tolerant rice cultivars. In this section, we briefly discussed the MAS and its significance in prospective research studies regarding plant breeding.

### 3.1 Marker-assisted crop breeding

**Figure 6:**
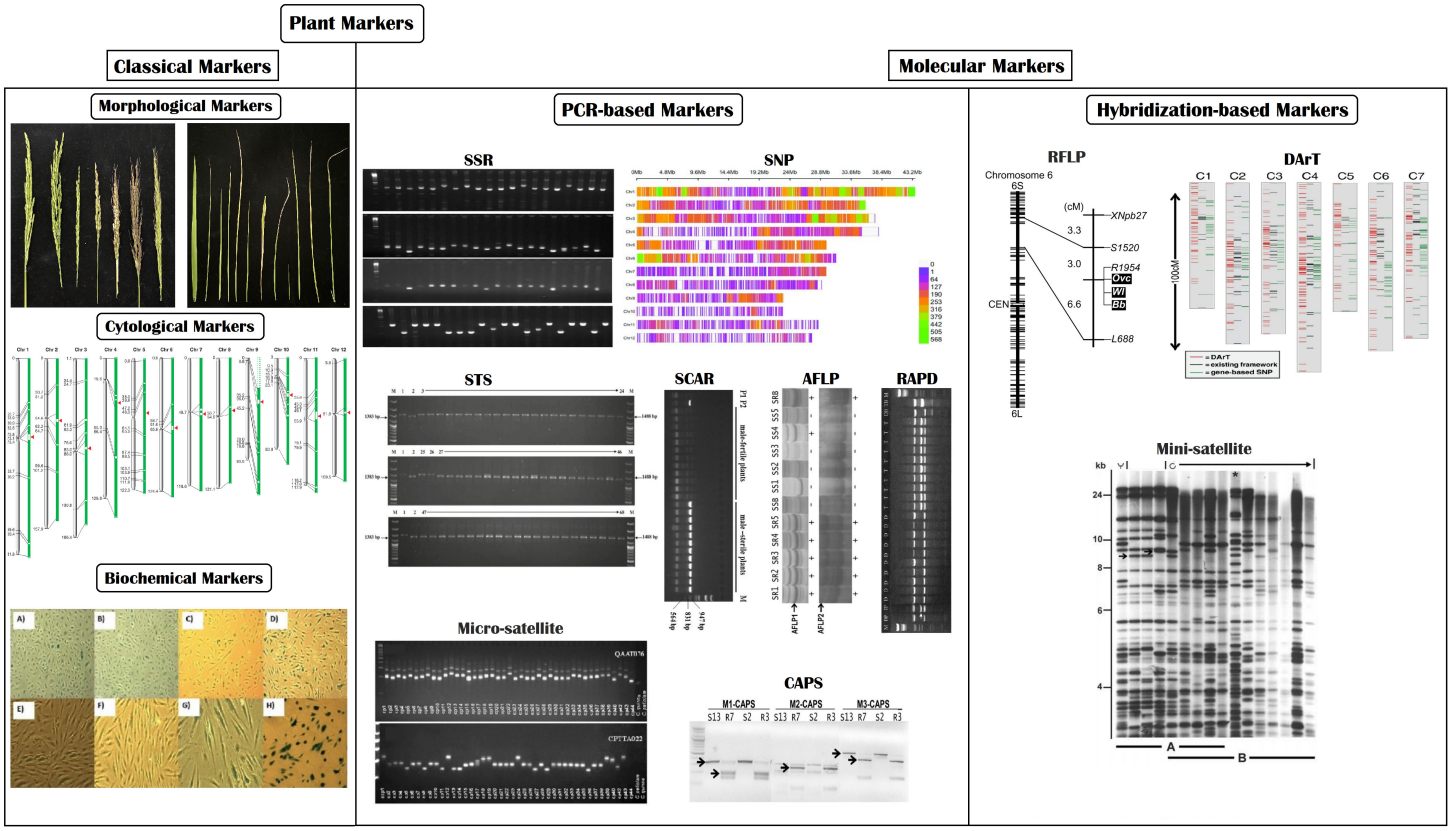
Pictorial illustration of commonly used plant markers.

In recent times, marker-assisted breeding approaches have been widely utilized, far better than conventional breeding techniques. It comprises multiple breeding techniques, such as identifying and mapping QTLs/genes and direct/indirect selection of genetic materials (Singh and Singh, 2015). Plant biomarkers are categorized into two major groups (1) classical markers and (2) molecular markers. Classical markers include morphological, cytological, and biochemical markers, whereas molecular markers include polymerase chain reaction (PCR) and hybridization-based molecular markers (**Figure 6**) (Joshi et al., 2016; Nadeem et al., 2017). Each type of marker has its own set of advantages and disadvantages, briefly stated in **Table 2**.

**Table 2:** Merits and demerits of commonly used markers.

Markers	Merits	Demerits	References
(1) Classical Markers			
**Morphological**	Inexpensive & affordable Simple to use Visual observation	Lower polymorphic Influenced by abiotic factors Altered with varying growth phases	(Eagles et al., 2001)
**Cytological**	Useful in chromosome identification & genetic linkage-mapping	Expensive High skill required	(Jiang, 2013; Nadeem et al., 2017)
**Biochemical**	Economically affordable Simple to use	Lower polymorphic Influenced by abiotic factors	(Mondini et al., 2009)
(2) PCR-Based			
**SSRs**	Lesser DNA quantity needed Higher rate of reproducibility Co-Dominant marker	Expensive development Homoplasy risk Null alleles	(Kalia et al., 2011; Dhingani et al., 2015)
**SNPs**	Prior Sequencing not required Higher rate of reproducibility Co-Dominant marker	Expensive development	(Kundan et al., 2014)
**STSs**	Highly Robust Higher rate of reproducibility Transferable	Specie-specific marker development	(Cato et al., 2001)
**AFLPs**	Highly polymorphic Dependable(Eagles et al., 2001) Higher rate of reproducibility	Dominant marker Highly pure DNA with more quantity required Complex	(Ridout and Donini, 1999; Frisvad et al., 2005)
**RAPDs**	Simple in use Lesser DNA quantity required Highly polymorphic	Lower rate of reproducibility Dominant marker Pure DNA needed Non-specific locus	(Adhikari et al., 2017)
(3) Hybridization-Based			
**RFLPs**	Co-dominant marker Prior sequencing is not required Transferable Locus-specific	Higher DNA quantity required Costly & Time-consuming Restricted polymorphism	(Adhikari et al., 2017)
**DArTs**	Economically affordable Highly polymorphic Prior sequencing is not required Higher rate of reproducibility	Expensive development Dominant marker	(Wenzl et al., 2004)
**Mini-satellite (VNTRs)**	Highly polymorphic Easy to map	Non-transferable Complex	(Marwal et al., 2014)

### 3.2 Classical markers

Classical markers are employed in various plant-breeding approaches but are not widely used because of certain limitations. Here we briefly described their potential use and limitations.

#### 3.2.1 Morphological markers

This kind of classical markers phenotypically distinguish plant growth and development characteristics such as plant height, flower color, seed structure, growth habit under abiotic stress, and other agronomic features (Karaköy et al., 2013). It is a cheaper and easy way to mark prominent characteristics of a specific crop plant. Though plant breeders use morphological markers in their breeding programs for cereal crops (i.e., rice, wheat, sugarcane), it has certain constraints as they are easily affected by different biotic/abiotic factors and crop growth stages (Eagles et al., 2001).

#### 3.2.2 Cytological markers

Cytological markers correspond to variations in the number, size, shape, banding pattern, position, and order of chromosomes (Nadeem et al., 2017). These markers provide a solid foundation for creating genetic linkage mapping and identifying normal and mutated chromosomes (Jiang, 2013; Xu et al., 2015).

#### 3.2.3 Biochemical markers

Multiple enzymes encoded by various genes and performing similar functions are called isozymes or biochemical markers (Bailey, 1983). These markers are used to compute the genetic frequencies of various genes and subsequently detect the genetic diversity in populations (Mateu-Andrés and De Paco, 2005). Like morphological markers, isozymes are also easy to use and cost-effective. Their efficiency is also influenced by environmental stress and different plant growth stage (Mondini et al., 2009).

#### 3.2.4 Molecular markers

The DNA fragments or gene sequences representing a particular locality in the genome are termed molecular markers (Semagn et al., 2006). Molecular markers are a significant advancement in modern plant breeding (Kebriyaee et al., 2012). Molecular markers are classified on the basis of their pattern of gene identification and their response to certain related traits. They are categorized as PCR- and hybridization-based molecular markers (Joshi et al., 2016). The PCR-based molecular markers are further categorized into microsatellite or SSR (simple sequence repeat), SNP (single nucleotide polymorphism), STS (sequence-tagged site), SCAR (sequence characterized amplified region), AFLP (amplified fragment length polymorphism) and RAPD (randomly amplified polymorphic DNA), and CAPS (cleaved amplified polymorphic sequence). In contrast, markers based on hybridization consist of minisatellites, RFLP (restriction fragment length polymorphism), and DArT (diversity array technology) markers (Gouda et al., 2020; Adhikari et al., 2017). The most used molecular markers include AFLP, RFLP, RAPD, SSR, SNP, and other microsatellites. The use of molecular markers also differs from species to species. The five most critical factors for selecting a marker are reliability, high parental polymorphism, high-quality DNA, expert marker assay, and affordability (Mackill and Ni, 2008).

Currently, molecular markers are popular because of their ability to be used in any plant part and at any phase of development. Further, they have been easily created in large numbers, with no sensitivity due to environmental stimuli (Singh, 2017; Marwal and Gaur, 2020). Molecular markers are employed to investigate the genetic make-up of a particular plant at the molecular level. In each group of plants, markers and genes are discovered within proximity on the same chromosome (Grover and Sharma, 2016; Marwal and Gaur, 2020). To quantify the closeness, a genetic linkage map may be constructed based on how far the markers are from a particular gene. This genetic linkage map was used to investigate the associative relationships between significant traits and genes/QTLs, which enables to access desirable genes/QTLs through the MAS technique (Panda et al., 2021; Singh, 2017). As a substitute for the selection of phenotypic traits, MAS attributed DNA markers affiliated with the target locus. Through the application of MAS, it is possible to relate DNA markers to highly significant features including disease resistance, abiotic/biotic stress tolerance, and agronomic traits (Sahebi et al., 2018; Oladosu et al., 2019). Mas-ud et al. (2022) used molecular markers to test 110 rice genotypes for their ability to withstand drought. They discovered 20 genotypes with varying degrees of drought resistance, from extreme to moderate degrees. Similarly, Roy et al. (2021) evaluated 30 rice varieties, among which 27 were identified as tolerant to drought stress. In brief, MAS is considered the cheaper and speedy route to develop climate-resilient rice cultivars (Dixit et al., 2020; Adjah et al., 2022). Therefore, the role of molecular markers is very important in the determination of genetic diversity, gene mapping and their utilization in breeding programs for the sake of the development of new drought-resilient cultivars.

### 3.3 Major steps of MAS

MAS is carried out by employing various kinds of genetic/molecular markers. MAS consists of the following major steps: (1) selection of parent plants with diverse origins, (2) development of population for mapping purposes (recombinant inbred lines-RILs, nearly isogenic lines-NILs and backcrosses-BCs used for F_2_ populations), (3) DNA extraction, (4) selection of suitable markers for QTL/gene mapping (i.e., RFLP, AFLP, SSR, RAPD, SNPs etc.), (5) phenotyping via correlation with morphological attributes, (6) construction of genetic-linkage pattern, (7) QTL/gene detection (genotyping), (8) validation, (9) and cloning. In this section, rather than detailing the earlier procedures, we thoroughly explored the various QTLs and genes associated with drought tolerance.

#### 3.3.1 QTLs associated with drought tolerance

The QTL mapping for drought-related attributes has been broadly investigated in various cereal crops, including rice (Dixit et al., 2017; Dixit et al., 2020). Vikram et al. (2011) reported that identifying QTLs associated with tolerance expressions is very useful in the success of drought research screening programs. Until now, several QTLs have been identified which are directly or indirectly linked with morpho-physiological and growth parameters and are widely used for selecting tolerant genotypes for rice plants (**Table 3**) (Dixit et al., 2017; Vinod et al., 2019). Gomez et al. (2006) identified 24 QTLs linked with morpho-physiological attributes under drought stress. Those QTLs were found for the following growth and yield attributes: 1 for panicle length, 5 for leaf tolling, 4 for leaf drying, 3 for days to half flowering (50%), 5 for plant height, 1 for straw yield, and 3 for grain yield. Since *DTY1.1* (mapped on chromosome 1), for rice plant height, was the firstly documented QTL in rice with a consistent effect in drought-tolerant elite varieties for improving grain yield, therefore the *DTY* series of QTLs is well-known and of great significance (Vikram et al., 2011). The *DTY3.1* (mapped on chromosome 3) is responsible for the maximum number of filled panicles under drought stress, significantly impacting rice grain yield (Mohd Ikmal et al., 2023). Barik and his team mapped 5 QTLs associated with morpho-physiological attributes of rice under drought stress. It includes *LR9.1* (mapped on chromosome 9), which regulates the leaf rolling, *LD9.1* (mapped on chromosome 9) for leaf drying, *SF9.1* (mapped on chromosome 9) for spikelet fertility, *RWC9.1* (mapped on chromosome 9) for relative water content, and *HI9.1* (mapped on chromosome 9) for harvest index, respectively, under water-deficit drought stress at the reproductive stage (Barik et al., 2022). Bernier et al. (2009) examined the *QTL12.1* (mapped on chromosome 12) for its impact on rice grain yield in water-deficit conditions in India. They concluded that *QTL12.1* has no impact under well water conditions, but its expression augmented up to 40% with increased drought intensity. Numerous studies have investigated various QTLs associated with drought tolerance in different rice lines such as *DTY 2.1, DTY2.2, DTY12.1* (for rice grain yield under drought, located on chromosome 2) (Dixit et al., 2012; Dixit et al., 2014), *DTHI2.3* (for harvest index under drought, located on chromosome 2) (Lin et al., 2007), *DTY6.1* (for enhancing grain yield under drought, located on chromosome 6) (Venuprasad et al., 2007), *DRL2.1* (for root length, located on chromosome 2) (Iqbal et al., 2021), and *DTY6.3* (for grain yield, located on chromosome 6) (Yadav et al., 2019). *DRO1* (located on chromosome 1) is a QTL associated with deeper root penetration under drought stress and enhances the yield potential in rice crops (Uga et al., 2013). Shamsudin and their colleagues identified three drought-yield QTLs (i.e., *DTY2.2,3.1* and *12.1*) in Malaysian cultivar MR219 under drought conditions using the MAS-pyramiding technique (Shamsudin et al., 2016). Later successfully developed a high yielding drought tolerant rice variety with a potential of 1500 kg/ha. Past studies identified the number of QTLs having their role in various biochemical and physiological processes (Barik et al., 2022); but wasn’t discovered associated genes with the same pace due to poor phenotypic impact and weaker mapping resolution (Singh et al., 2012; Bin Rahman and Zhang, 2016). Several studies have found a diverse range of molecular markers linked to these QTLs and used to screen new rice genotypes for drought tolerance.

**Table 3:** Rice QTLs/Genes associated with drought tolerance.

QTLs	Trait/Function	Reference	Genes/TFs	Trait/Function	References
***qLR8.1*** ***qLR9.1*** ***qDLR8.1***	Leaf rolling	(Barik et al., 2019; Dixit et al., 2012; Lin et al., 2007)	*OsGRF6* *AtMYB60* *OsPYL*	Adjust shape and architecture Control stomatal aperture Regulate stomatal closure	(Cominelli et al., 2005; Kim et al., 2014; Kim et al., 2020; Zhou et al., 2010)
***qLD9.1*** ***qLD12.1***	Leaf drying	(Barik et al., 2019)	*OsTPS1*	Proline & soluble sugar accumulation	(Li et al., 2011)
***qRWC9.1***	Relative water content	(Barik et al., 2019)	*AtDREB1A*	Accumulation of osmoprotectants	(Ravikumar et al., 2014)
***qDTR8***	Transpiration rate	(Ramchander et al., 2016)	*DsM1*	ROS scavenging	(Ning et al., 2010)
***qSF9.1***	Spikelet fertility	(Barik et al., 2019)	*OsbZIP71* *AP37* *OsNAC1*	Enhance seed setting Increased seed filling Improved spikelet fertility	(Hu et al., 2006; C. Liu et al., 2014; Oh et al., 2009)
***qPH1.1***	Plant height	(Trijatmiko et al., 2014)	*OsbZIP23* *OsLEA3-1* *OsWRKY47*	Enhance grain yield Lower yield reduction	(Xiao et al., 2007; Xiang et al., 2008)
***qPL-9*** ***qGY3.1*** ***qDTY2.3*** ***qHGW1*** ***QHI9.1***	Panicle length & number Grain yield Total dry matter Harvest Index	(Lanceras et al., 2004; Sandhu et al., 2014; Sellamuthu et al., 2015; Barik et al., 2019)	*OsDRO1* *OsNAC10* *OsNAC5* *OsDREB2B*	Deep elongated roots Root diameter Number of roots	(Jeong et al., 2013; Uga et al., 2013)

#### 3.3.2 Genes associated with drought tolerance

Under Drought stress, rice plants exhibited a number of up/down regulated genetic expressions. It includes 5000 up-regulated and 6000 down-regulated expressions (Joshi et al., 2016). These genetic expressions are related to drought stress signaling, membranous transport, and transcriptional control during cellular metabolism (Kim et al., 2020). Many genes expressed under drought lie in ABA-dependent or independent regulation mechanisms (Gupta et al., 2020). Fu et al. (2017) stated that *OsJAZ1* reduces drought tolerance via modulating ABA signaling, which coordinates plant responses to drought stress in rice. Root morphological responses to drought stress were carried through over-expressions of *OsDREB2B*, *CYP735A*, and *OsDREB1F* (Kim et al., 2020). Uga et al. (2013) revealed that *DRO1* is the responsible gene for root elongation and deeper penetration under water-limited conditions. Genetic expressions of *OsCPK9* increase plant tolerance to drought by enhancing stomatal closure and improving osmoregulation (Wei et al., 2017). Drought response at the vegetative stage is facilitated through the induction of *OsNAC10* (Jeong et al., 2013). The gene expressions of *OsMIOX* during severe drought stress prevent oxidative damage by activating ROS-scavenging enzymes (Duan et al., 2012). **Table 3** Shown the list of such genes and their related functions for rice drought tolerance. Through MAS, these novel genotypes can be utilized in conventional and modern breeding programs to create rice varieties that are more compatible with drought conditions. The International Rice Research Institute has conducted most marker-assisted breeding trials in the last decade for developing drought-resistant rice varieties(Kumar et al., 2017; Sandhu and Kumar, 2017).

## 4 Conclusion and prospects

Rice plants exhibit critical behavior in stressful conditions, requiring proper management. Tolerance strategies, pathways, and mechanisms in drought have long been debated in scientific research. Different studies have been conducted to explore the causes of drought stress, and strategies have been devised to mediate the plant responses. We have elaborated on the rice-plant responses to drought stress at the morphological, physiological, biochemical, and molecular levels. Further, we discussed the role of modern breeding techniques (such as MAS) in increasing the pace of ongoing breeding programs for varietal development. The significance of available genetic resources (i.e., genotypes, QTLs, and other genetic make-ups) has been explained concerning their phenotypic characteristics under drought. Despite the number of studies, scientific research has not succeeded in figuring out the ideal plant responses in drought. The role of genes and QTLs is needed to be further explored because of their controlling potential in plant acclimation responses during stress. The discovery of potential genes/QTLs responsible for drought acclimation responses is a real quest for crop scientists in the current era of changing climate.

A broader understanding of the genetic basis of agricultural attributes in many crops has recently been accomplished because of advancements in molecular marker-assisted technologies. Modern research focuses on managing genotypes to demonstrate the effect on phenotype and introgressing them into high-yielding varieties. The importance of molecular markers and hereditary material needs to be further investigated in the near future to attain better opportunities to grow the plant under stressful environments. Detailed genome-wide association studies are needed to overcome the loopholes in markers-assisted breeding. In addition to this, modern molecular markers are quite expensive and required skilled persons for effective utilization. Along with the availability of low-cost molecular markers, it is critical to conduct training programs in research institutes to equip scientists and researchers with modern techniques and skills for efficient resource utilization for the development of climate-smart varieties. Economic conditions’ favorability will stimulate the application of such gene-identifying/editing tools and techniques in a pragmatic way.

## Author contributions

MH and WS conceived the concept of the review and prepared an outline of the review. MH and ZQ compiled the literature and wrote the different sections. YY and LY. aided in designing figures and arranging references. ND and TH provided technical assistance and editing support.
